# Case report of a traumatic rectal neuroma

**DOI:** 10.1093/gastro/gov023

**Published:** 2015-06-19

**Authors:** Thomas Curran, Vitaliy Poylin, Robert Kane, Anna Harris, Jeffrey D. Goldsmith, Deborah Nagle

**Affiliations:** ^1^Department of Surgery, Division of Colorectal Surgery, Beth Israel Deaconess Medical Center, Boston, MA, USA;; ^2^Department of Radiology, Beth Israel Deaconess Medical Center, Boston, MA, USA and; ^3^Department of Pathology, Beth Israel Deaconess Medical Center, Boston, MA, USA

**Keywords:** Traumatic rectal neuroma, transanal endoscopic microsurgery

## Abstract

Traumatic neuroma is a well-recognized complication of lower extremity amputation, yet has also been noted to occur elsewhere. We report a clinical case and English-language literature review of traumatic rectal neuroma, a well-known pathologic entity not previously reported in this anatomic location.

## Introduction

Traumatic neuroma is a well-recognized complication of lower extremity amputation, yet has also been noted to occur elsewhere. Additional locations include the head and neck in the setting of tooth extraction or upper extremity following trauma to the radial nerve or brachial plexus [1, 2]. Intra-abdominally, there have been more than 80 described cases of common bile duct traumatic neuroma following biliary surgery. We report a clinical case and English language-literature review of traumatic rectal neuroma, a well-known pathologic entity not previously reported in this anatomic location.

## Case report

**Figure 1.**
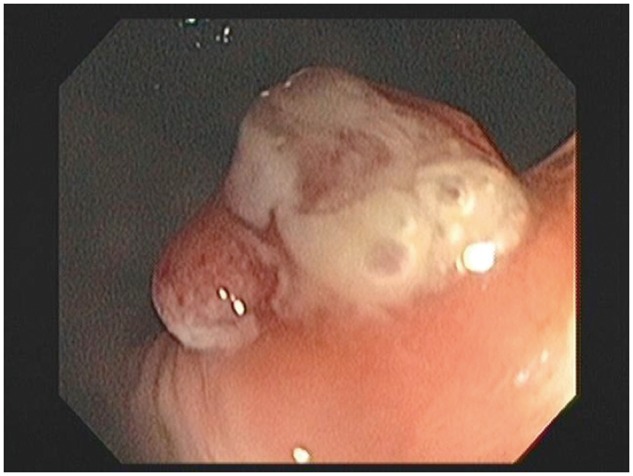
Colonoscopic view of incompletely excised 8mm distal rectal polyp.

The patient is a 53-year-old gentleman who came to the attention of our colorectal surgery clinic following incomplete colonoscopic removal of an 8mm distal inflammatory rectal polyp (Figure 1). His past medical history was unremarkable with the exception of irritable bowel syndrome and history of a benign rectal polyp removed endoscopically six years prior. On presentation, the patient complained of peri-anal pruritis but denied rectal pain, hematochezia and dyschezia. Physical examination demonstrated a firm lobulated, anteriolateral nodule 3cm above the levators at the site of the incompletely excised polyp.

**Figure 2.**
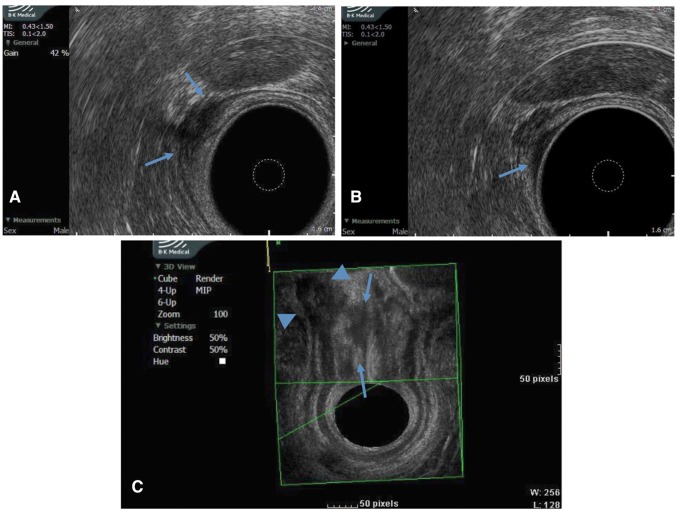
Endorectal ultrasound of rectal lesion showing submucosal involvement. (A) Axial view with arrows denoting mural component in muscularis propria; (B) Axial view with arrow demonstrating mucosal component; (C) 3D reconstruction showing mucosal (arrows) and muscular wall (arrowheads) components.

After ten months lost to follow up, biopsy specimens of the lesion showing chronic inflammation were obtained in the setting of an attempted transanal excision that was aborted secondary to difficult anatomy. Subsequent endorectal ultrasound revealed a complex lesion just proximal to the internal sphincter. The lesion demonstrated an unusual bifid appearance with contiguous components extending into the submucosa proximally and the muscularis propria distally (Figure 2). Transanal endoscopic microsurgery was then successfully employed to resect the lesion, and the patient returned home on postoperative day two following an uneventful course.

**Figure 3.**
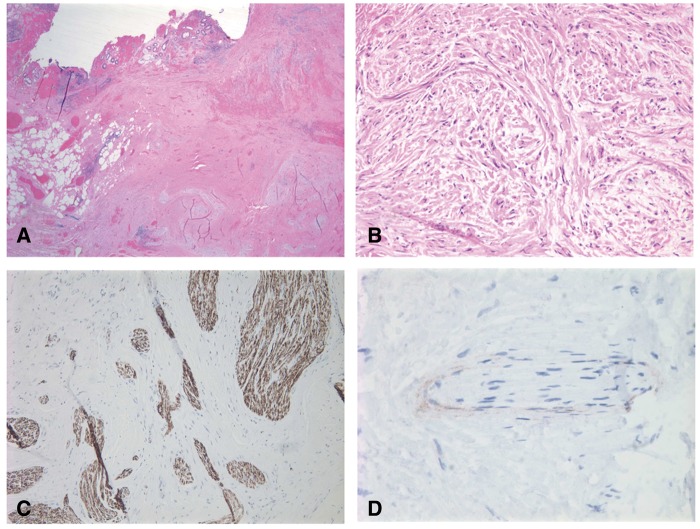
Pathology images of traumatic rectal neuroma. (A) Low power view. Note overlying mucosa with hemorrhage and ulceration. Submucosa and muscularis propria are involved by a proliferation of nerves growing in a haphazard arrangement. (B) High power view of nerve bundles. (C) S100 and neurofilament highlight the nerve bundles. (D) Epithelial membrane antigen highlights the perineurium.

Pathology demonstrated a traumatic neuroma, 1cm in largest dimension, with negative margins within a specimen measuring 3.9 × 3.6 cm overall. The presence of S-100, neurofilament and epithelial membrane antigen on immunohistochemical stain confirmed the diagnosis (Figure 3). No further treatment was required for this benign lesion. There was no evidence of recurrence by colonoscopy or physical exam at four years.

## Discussion

Traumatic neuromas are non-neoplastic proliferative lesions that may manifest at the proximal end of a nerve that is severed, partially transected or injured via either traumatic or surgical means [1, 2]. These fibroinflammatory lesions arise in two varieties according to the type of insult sustained by the peripheral nerve. Terminal neuromas arise at the proximal end of a partially or fully transected peripheral nerve, while spindle neuromas develop in a non-disrupted nerve that is subject to repeated traumatic irritation [3].

When re-approximation of an injured nerve is not possible, the subsequent process of inflammation and regeneration may engender a non-encapsulated, disorganized tangle of axons, Schwann cells, endoneurial cells and perineurial cells surrounded by fibroblasts [2]. Aberrant neuro-electrical activity within this constellation of cells may then manifest as neuropathic pain, the most common presenting symptom of traumatic neuroma.

Management of traumatic neuroma in the head, neck or extremities is typically conservative, initially consisting of steroid injections, nerve stimulation or injectable sclerosants [2]. Approximately half of patients remain refractory to conservative therapy and require surgical resection for definitive management. Management of common bile duct traumatic neuroma following biliary surgery is predicated upon relieving biliary obstruction by either endoscopic or surgical means [4].

Although traumatic neuroma is a well-described clinical entity, review of the English- language literature demonstrates no previously reported cases of traumatic neuroma located in the rectum. Accordingly, the following discusses one such case that was remarkable for its unusual presentation and diagnostic difficulty.

While traumatic neuroma typically presents with pain, the aforementioned patient was asymptomatic from his lesion. These lesions typically arise from peripheral somatic nerves in the setting of amputation, yet because the rectum lacks somatic innervation, it is fitting that our patient was asymptomatic. Similarly, traumatic biliary neuroma patients present with obstructive jaundice secondary to mass effect but without neuropathic pain, given the insensate nature of the common bile duct [4, 5].

Regarding the cause of this traumatic rectal neuroma, the traumatic insult responsible for development of the lesion is unclear. The patient suggested a remote sports injury to his lower back as a source of trauma, but he denied anopenetrative intercourse or any other type of direct rectal trauma. It should be noted that the patient suffered from obsessive-compulsive disorder with a propensity for repeated external wiping, although he denied any internal rectal manipulation, making repeated rectal irritation less likely. More likely is that the patient’s lesion was the result of trauma to submucosal Meissner’s plexus fibers or Aurbach’s plexus fibers during one of his two endoscopic polypectomies. Traumatic neuromas have been seen to develop within one to 12 months following nerve trauma, and this would fit the time interval from the patient’s colonoscopy to the time he had endoscopic ultrasound confirming the presence of an intramural lesion [3].

Given the patient’s family history of colon cancer and a personal history of a rectal polyp, neoplasm was a significant consideration for his rectal mass. Though the patient’s initial polypectomy demonstrated an inflammatory polyp at this site, the presence of a mass within the muscularis propria, seen on endoscopic ultrasound in the setting of non-diagnostic core biopsies, left little option other than excisional biopsy. Recommendations for management of traumatic rectal neuroma in future cases would be highly dependent on the specific considerations for the particular patient related to the certainty of diagnosis as well as anatomic location and amenability for surgical resection.

In conclusion, traumatic rectal neuroma is a rare pathologic entity that should be included in the differential diagnosis for a rectal mass found in the setting of previous rectal trauma or polypectomy. These represent a benign process, the management of which should be individualized based on the clinical issues facing each specific patient.

*Conflict of interest statement*: none declared.

## References

[1] Abreu E Aubert S Wavreille G Peripheral tumor and tumor-like neurogenic lesions. Eur J Radiol 2013;82:38–50. 21561733 10.1016/j.ejrad.2011.04.036

[2] Murphey MD Smith WS Smith SE From the archives of the AFIP. Imaging of musculoskeletal neurogenic tumors: radiologic-pathologic correlation. Radiographics 1999;19:1253–80. 10489179 10.1148/radiographics.19.5.g99se101253

[3] Woertler K Tumors and tumor-like lesions of peripheral nerves. Semin Musculoskelet Radiol 2010;14:547–58. 21072731 10.1055/s-0030-1268073

[4] Ueno Y Ikeda K Maehara M Traumatic neuroma of the bile duct. Abdom Imaging 2008;33:560–2. 18360736 10.1007/s00261-007-9318-x

[5] Iannelli A Fabiani P Karimdjee BS Traumatic neuroma of the cystic duct with biliary obstruction. Report of a case. Acta Gastroenterol Belg 2003;66:28–9. 12812146

